# Utility of passive malaria surveillance in hospitals as a surrogate to community infection transmission dynamics in western Kenya

**DOI:** 10.1186/s13690-018-0288-y

**Published:** 2018-07-26

**Authors:** Anthony Kapesa, Eliningaya J. Kweka, Guofa Zhou, Harrysone Etemesi Atieli, Erasmus Kamugisha, Humphrey D. Mazigo, Sospatro E. Ngallaba, Andrew K. Githeko, Guiyun Yan

**Affiliations:** 1 Climate and health laboratory, Centre for Global Health Research, Kenya Medical Research Institute, P.O. Box 1578, Kisumu, Kenya; 2 Department of Community Medicine, School of Public Health, Catholic University of Health and Allied Sciences, P.O Box 1464, Mwanza, Tanzania; 3 Department of Medical Parasitology and Entomology, School of Medicine, Catholic University of Health and Allied Sciences, P.O Box 1464, Mwanza, Tanzania; 4 Department of Biochemistry and molecular Biology, School of Medicine, Catholic University of Health and Allied Sciences, P.O. Box 1464, Mwanza, Tanzania; 5 Division of Livestock and Human Health Disease Vector Control, Tropical Pesticides Research Institute, P.O. Box 3024, Arusha, Tanzania; 6 Program in Public Health, University of California, Irvine, CA 92697 USA

**Keywords:** Outpatient department (OPD), Malaria blood slide positivity, School age children

## Abstract

**Background:**

Malaria continued to be the major public health concern in sub-Sahara Africa, thus for better planning of control activities, periodic surveillance of both clinical and asymptomatic cases remains important. However, the usability of routinely collected malaria data in Kenyan hospitals as a predictor of the asymptomatic malaria infection in the community amidst rapid infection resurgence or reduction in different areas of disease endemicities remains widely unstudied. This study was therefore aimed to evaluate the utility of passive surveillance of malaria in health facilities as a proxy of infection transmission of the surrounding community in different transmission intensities.

**Methods:**

Prospective multiple cross-sectional surveys were done in three villages in western Kenya. Monthly asymptomatic malaria positivity among school children, number of outpatient (OPD) confirmed malaria cases and abundancy of indoor resting malaria vectors were surveyed from June 2015 to August 2016. Community surveys on antimalarial drug use among adults and children were also done. Detection of malaria parasitaemia was done using thick and thin Giemsa stained blood slide microscopy for both clinical and school participants. A questionnaire was used to collect information on self-use of antimalarial drugs from randomly selected households.

**Results:**

The overall OPD blood slide positivity from all study sites was 26.6% (95%CI 26.2–27.0) and highest being among the 5–14 years (41.2% (95% CI 40.1–42.3). Asymptomatic malaria positivity among the school children were 6.4% (95%CI 5.3–7.5) and 38.3% (95%CI 36.1–40.5) in low and high transmission settings respectively. A strong correlation between overall monthly OPD positivity and the school age children positivity was evident at Marani (low transmission) (rho = 0.78, *p* = 0.001) and at Iguhu (Moderate transmission) (rho = 0.61, *p* = 0.02). The high transmission setting (Kombewa) showed no significant correlation (rho = − 0.039, *p* = 0.89).

**Conclusion:**

Hospital malaria data from low and moderate malaria transmission predicted the infection transmission dynamics of the surrounding community. In endemic sites, hospital based passive surveillance didn’t predict the asymptomatic infection dynamics in the respective community.

**Electronic supplementary material:**

The online version of this article (10.1186/s13690-018-0288-y) contains supplementary material, which is available to authorized users.

## Background

Malaria infection in tropical and sub-tropical countries continued to be a major public health concern [1]. In 2015 alone, about 429,000 deaths were due to malaria globally, 92% of them were in Africa [1]. Approximately 70% of Kenyan population were at risk of malaria infection with an immense threat in the coast and lake endemic areas of the country [2]. The introduction of intense distribution and use of interventions against malaria has emanated to a decrease of both mortality and morbidity [3, 4]. However, despite the intensive use of malaria intervention measures, some areas in Kenya have witnessed persistence of asymptomatic infection and clinical cases [5–7]. This scenario has been as well observed elsewhere [8–10].

Monitoring of malaria transmission dynamics require both local and nationwide surveillance mechanisms. Malaria control programs conduct regular indicator surveys for nationwide planning and decision making [2, 11]. The Kenya malaria control strategy (2009–2018) recommend that all malaria indicators to be local, routinely monitored and evaluated [12]. The local health authorities were therefore required to have the capacity to detect and respond in a timely manner to malaria outbreaks [12]. Surveillance of malaria in schools is one of the recommend approaches to monitor local transmission dynamics [13]. In western Kenya, the asymptomatic malaria positivity rate among primary school pupils has been correlated with that of the respective communities [14]. Moreover, asymptomatic blood slide positivity rates among under-five children or pregnant mothers attending antenatal clinic in Tanzania correlated with the overall outpatient blood slide positivity rates [15]. The introduction of long lasting insecticide treated nets, artemisinin based combination therapy and indoor residual spray in sub-Saharan Africa has resulted to a significant reduction malaria morbidity and mortality [1]. Active surveillance of asymptomatic infections and hospital based passive surveillance have therefore been used to monitor the impact of the disease in Kenya [2, 5, 16, 17]. However, accuracy of the passive malaria surveillance in hospitals as a predictor of community transmission dynamics in the context of different infection intensities has not been widely contemplated. In the midst of a changing infection dynamics, exploration of the reliability of passive malaria surveillance in health facilities as a predictor of infection transmission rate in surrounding communities is therefore imperative. This study therefore aimed to evaluate the utility of the routine passive malaria surveillance in health facilities as a proxy of the infection transmission dynamics of the surrounding catchment population.

### Study area

**Fig. 1:**
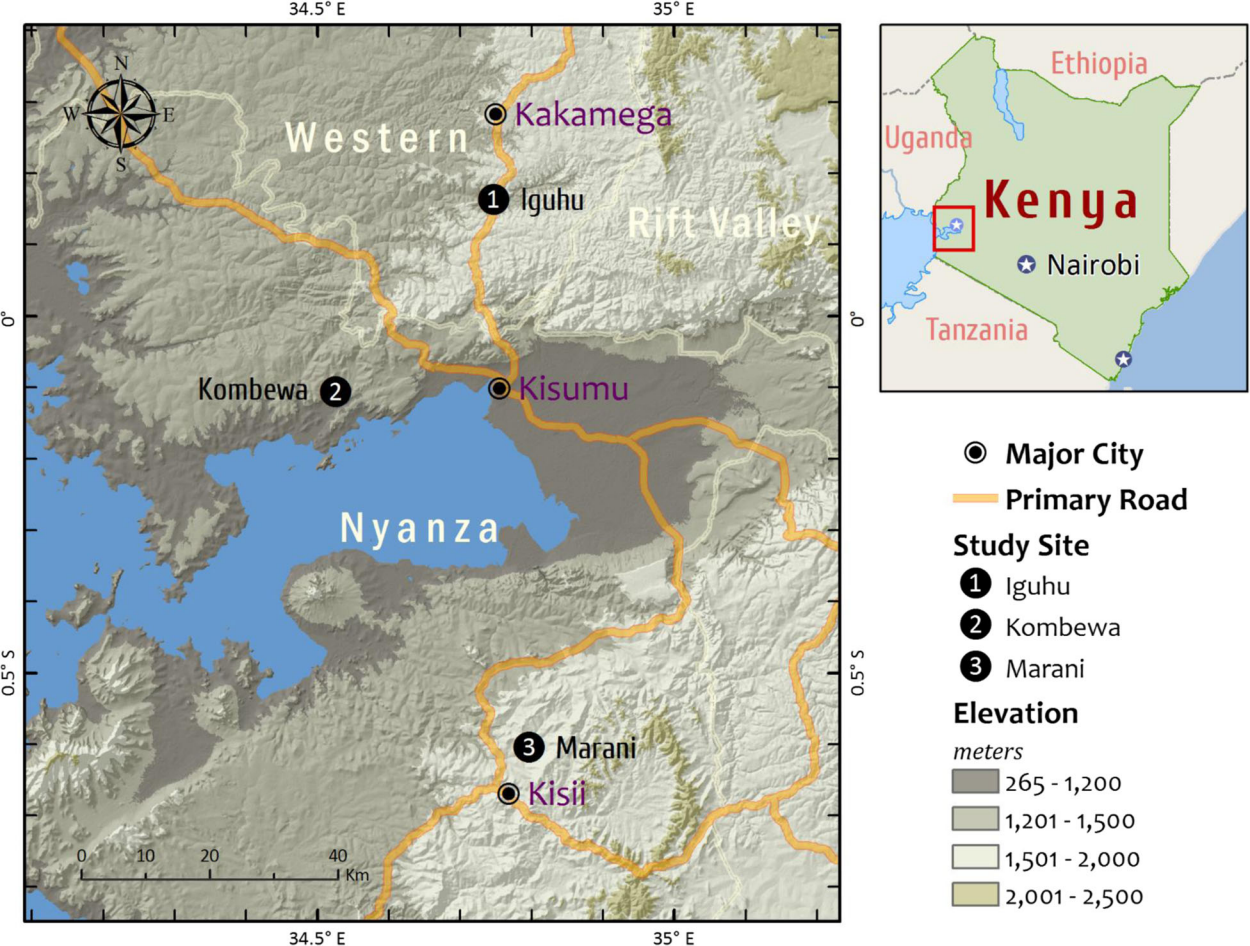
Map of showing the location of the three study sites in areas with different malaria infection transmission intensity in western Kenya

This study was performed in three villages with different altitude, topography and malaria transmission intensity (Fig. 1). The first study area was Kombewa located in Kisumu County, a lowland setting with hyperendemic transmission intensity [16] positioned at 34°30′E, 00°07′N; 1150–1300 m above the sea level (ASL). This area had approximately 23,000 inhabitants occupying a flat land with vast malaria breeding habitats especially during the rainy season. Kombewa has one government owned hospital (Kombewa County Hospital) where this study was conducted together with some selected government primary schools located within the study area. Four schools were selected out of 21 at Kombewa, these schools were; Akonya, Diemo, Kamonye and Okode. The second study area was Iguhu which is a highland site positioned at 34°45′E, 00°10′N; 1430–1580 m ASL. This area has a moderate malaria transmission referred as mesoendemic [16]. The area had about 24,000 people with hills and wide valleys. Water drainage in valleys is not very fast thus leaving some stagnation of water in some areas which favours Anopheline breeding. Iguhu County Hospital and 2 selected primary schools (Ivonda and Iguhu) were the study sites for this area. This area has a total of twenty primary schools with one government owned hospital (Iguhu). Residents from this study area are also served by the nearby private hospital (Mukumu). The third study site was Marani located in Kisii County, the area represented a highland malaria ecology with epidemic prone malaria transmission [16] and located at 34°48′E, 00°35′S; 1540–1740 M ASL. The area had approximately 19,000 residents with valleys and elevations with high populations at top hills. The drainage system was made up of very narrow valleys which allows water to flow very fast resorting to few mosquito breeding habitat. The area had only one government owned hospital (Marani), this was also used as a study site. At Marani, school children attending Gesangora, Nyasaga and Kiraeni primary schools were used to determine the dynamics of asymptomatic malaria infection. All three areas experience generally short and long double-peak rainfall pattern starting from April to June, associated with increased malaria transmission, and a short rainy season starting from October to November. Generally the study areas have rainy and dry seasons and *Plasmodium falciparum* is the most prevalent malaria parasite transmitted mainly by *Anopheles gambiae* s.s., *An*. *arabiensis* and *An*. *Funestus* [16].

### Study design

This study involved multiple cross-sectional surveys conducted monthly from June 2015 to August 2016. Surveillance of asymptomatic malaria among primary school children, OPD confirmed malaria and indoor resting malaria vectors for 15 observations. Moreover community based cross-sectional surveys on the use and source of anti-malarial were done in June and December 2016. A snap shot additional study to determine accuracy of blood slide malaria microscopy examination was conducted at Iguhu hospital (moderate transmission site).

### Survellance of laboratory confirmed malaria

All patients attended the OPD and suspected to have malaria were examined by Giemsa stained blood slides and their results were recorded on daily basis on the specific registry as from June 2015 to August 2016. A total of 48,892 malaria suspected patients were examined by Giemsa stained blood slide in the three hospitals**.** A total of 17,231, 20,985 and 10,676 suspected patients were tested for malaria at Marani, Iguhu and Kombewa hospitals respectively.

### Sample size calculation

Surveillance of asymptomatic infection in schools required minimum of 150 school age children per month per study site. The sample size was based on the local prevalence asymptomatic malaria infection ranging from 6.2 to 47.1% [16]. A total of 30 randomly selected houses in each village were used to collect indoor resting vectors per site per month along with the school surveillance [18]. This study also assessed malaria self-treatment practice in households where a minimum of 250 household were visited per site per survey [2]. To determine accuracy of microscopic diagnosis of malaria in clinical setting, a total of 200 blood slides from one selected health facility were re-examined [19].

### Surveillance of asymptomatic malaria among school age children

Multiple cross-sectional surveys were conducted among school children attending nine primary schools in the study areas. A finger prick blood sample was obtained from each school child, thick and thin blood smears prepared, stained with Giemsa and examined using light microscope. Sum of 1795, 1972 and 2430 blood slides were collected and examined from Kombewa, Marani and Iguhu respectively as from June 2015 to August 2016.

### Survellance of indoor resting malaria vectors

A total of 30 randomly selected houses in each village were used to collect vectors per site per month as from June 2015 to August 2016. Pyrethrum Spray Catch (PSC) method as described elsewhere [18] was used to collect the indoor resting malaria vectors, morphological identification of vectors was performed using a dissecting microscope with an identification guide [20].

### Household survey of anti-malarial drug use

The survey involved 756 and 1500 randomly selected participants from households who had used anti-malarial drugs prior to the interview in June 2016 and December 2016 respectively. Using a structured questionnaire, information on the use of anti-malarial drug within 2 weeks prior to the interview and type of dispensing unit were inquired.

### Accuracy of malaria microscopic diagnosis

About 200 Giemsa stained thin and thick smear blood sides among malaria suspected patients from a hospital located in mesoendemic transmission setting were collected and re-examined by two qualified laboratory technicians at the Kenya Medical Research Institute in Kisumu. Malaria suspected patients were those with history of chills, body malaise, joint pain and other symptoms together with fever above 37.5^0^ C. Malaria suspected found with parasitemia were termed as clinical malaria cases. Malaria parasites (asexual form) and gametocytes were counted at each 200 white cell fields. Technicians who re-examined the blood slides were blinded of the hospital microscopy results and of each other’s results.

### Data analysis

The monthly and overall prevalence of malaria parasites per site among the school children and among the symptomatic patients were computed. The mean number of female Anopheline mosquitoes identified per house per night were calculated and compared between the villages. Variations of vector abundance was compared between the study sites using one way analysis of variance (ANOVA) and Tukey-Kramer post-hoc test. The frequency of household anti-malarial drug use per age group and per site was computed. Cohen’s kappa measurement of agreement was used to determine blood slide results concordance between hospital results and those re-examined at the research centre. Relationship between monthly asymptomatic malaria positivity among school children and clinical blood slide positivity was determined by using Spearman’s correlation test. Spearman’s correlation between abundance of indoor resting female Anopheline mosquitoes collected by PSC and the different clinical age groups malaria positivity was also done. Regression analysis between the abundance of indoor vectors and the clinical blood slide positivity rate was done to determine strength of association. Spearman’s correlation coefficient (rho) and regression analysis were used to determine strength of associations. The association strength was considered significant when the *p*-value was < 0.05. Strong correlation and modest correction were defined when the *p*-value was ≤0.001 and < 0.01 respectively. Graphic presentation of data, display of trend lines as well as predictor equations were computed. The correlation analyses were done by using SPSS® version 17 and determination of linearity computed using Microsoft excel® window 10.

## Results

### Hospital malaria positivity

The overall blood slide positivity (for 15 month observation) for all study sites was 26.6% (95%CI 26.2–27.0) and 27.4% (95%CI 26.9–27.9) for the first 12 months. The highest OPD malaria positivity rate was observed among children aged 5–14, with 45.3% (95% CI 42.8–47.8) and 44.5% (95%CI 42.8–46.2) at Kombewa and Marani hospital respectively (Table 1, Additional file 1). The lowest OPD positivity rates were 18% (95%CI 17.2–18.8) and 18.9% (95%CI 17.9–0.20) among the ≥15 at Iguhu and Kombewa respectively (Table 1, Additional file 1).

**Table 1:** Summary of number of malaria suspected patient who underwent blood slide examination as from June 2015 to August 2016

Study site	Characteristic of malaria blood slide				95% CI
	Age group	Blood slides examined	Total positive	Positivity (%)	
Marani	< 5	6425	1457	22.6	21.7–23.7
	5–14	3267	1453	44.5	42.8–46.2
	≥15	7539	1689	22.4	21.5–23.3
Iguhu	< 5	8811	2459	27.9	26.9–28.8
	5–14	3267	1182	36.2	34.5–37.8
	≥15	8907	1603	18.0	17.2–18.8
Kombewa	< 5	3754	1487	39.6	38.1–41.2
	5–14	1493	676	45.3	42.8–47.8
	≥15	5429	1031	18.9	17.9–20.0
Total		48,892	13,037	26.6	26.2–27.1

### Surveillance of asymptomatic malaria among school children

The blood slide positivity was consistently highest at the high transmission study area (Kombewa) with an overall prevalence of 38.3% (95%CI 36.1–40.5) and lowest at the low transmission setting (Marani) with 6.4% (95%CI 5.3–7.5) (see Additional file 2). The positivity rate within the first 12 months was 38.9% (95%CI 36.5–41.4) and 7.2% (95%CI 5.9–8.4) for Kombewa and Marani respectively.

### Surveillance of indoor resting malaria vectors

The mean density of indoor resting vectors (for 15 months observations) were 0.59 ± 0.22, 0.68 ± 0.08 and 1.01 ± 0.28 female anopheline mosquitoes per house per night at Marani, Iguhu and Kombewa respectively. Moreover, for the first 12 months the mean densities were 0.52 ± 28, 0.63 ± 0.15 and 0.98 ± 0.33 female anopheline mosquitoes per house per night at Marani, Iguhu and Kombewa respectively. There was significant variation of the monthly abundance of indoor vectors between the study areas (F = 3.66_,_ df _=_ 2, *p* < 0.05). Post hoc Tukey-Kramer test however revealed existence of a significant variation of indoor vectors only between Marani and Kombewa (see Additional file 2).

### Correlation between OPD malaria positivity and asymptomatic infection among school children

**Fig. 2:**
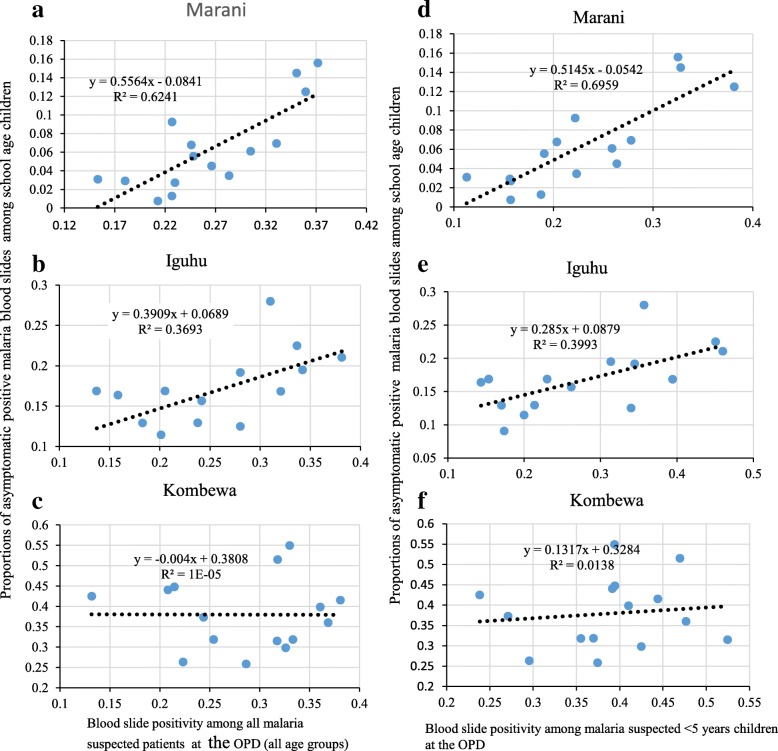
Correlation between monthly all age groups and <5 children OPD positivity with asymptomatic malaria positivity among school age children in western Kenya from June 2015 to August 2016 (**a**, **b**, **c** show correlation between monthly all age groups OPD malaria positivity and school age children asymtomatic malaria positivity; **d**, **e**, **f** show correlation between the <5 children OPD malaria positity and school age children asyptomatic malaria positivity)

**Fig. 3:**
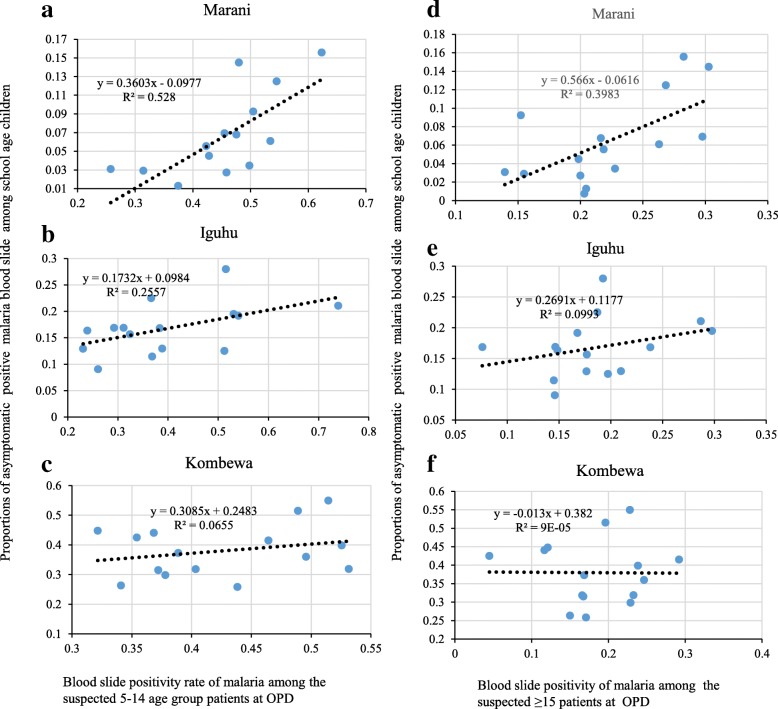
Correlation between monthly 5–14 and ≥ 15 OPD positivity with asymptomatic malaria positivity among school age children in western Kenya from June 2015 to August 2016 (**a**, **b**, **c** show correlation between OPD malaria positivity among the 5-14 years and asypmtomatic malaria positivity among school age children; **d**, **e**, **f** show correlation between OPD malaria positivity among the fifteen and above and asymptomatic malaria positivity among school age children)

Malaria positivity rate among suspected patients from selected health facility was correlated with asymptomatic malaria positivity among school children in respective areas. In highland settings, correlation between all age group OPD positivity and school children asymptomatic positivity was strongest at Marani (rho = 0.78, *p* = 0.001) and moderate at Iguhu (rho = 0.61, *p* = 0.02). However, the high transmission study site (Kombewa) had no significant correlation between overall OPD positivity and asymptomatic infection positivity among school children (rho = − 0.039, *p* = 0.89) (Fig. 2a, b, c). Regarding the under-five OPD positivity, this study observed a stronger correlation with the school children asymptomatic positivity at Marani (rho = 0.83, *p* = 0.000) than Iguhu (rho = 0.62, *p* = 0.01) but showed no correlation at Kombewa (rho = 0.12, *p* = 0.676) (Fig. 2d, e, f). Furthermore, the 5–14 age group and ≥ 15 age group OPD positivity at Marani significantly correlated with the school children asymptomatic infection positivity, rho = 0.75, p = 0.001 and rho = 0.61, *p* = 0.02 respectively (Fig. 3a, d). School children asymptomatic positivity at Iguhu showed neither significant correlation with the 5–14 OPD positivity (rho = 0.489, *p* = 0.06) nor the ≥15 OPD positivity (rho = 0.39, *p* = 0.16) (Fig. 3b, e). At Kombewa the 5–14 age group and ≥ 15 age group OPD positivity showed no correlation with school children asymptomatic positivity, rho = 0.11, *p* = 0.704 and rho = − 0.068, *p* = 0.81 respectively (Fig. 3c, f).

### Abundance of indoor resting malaria vectors and OPD positivity

**Fig. 4:**
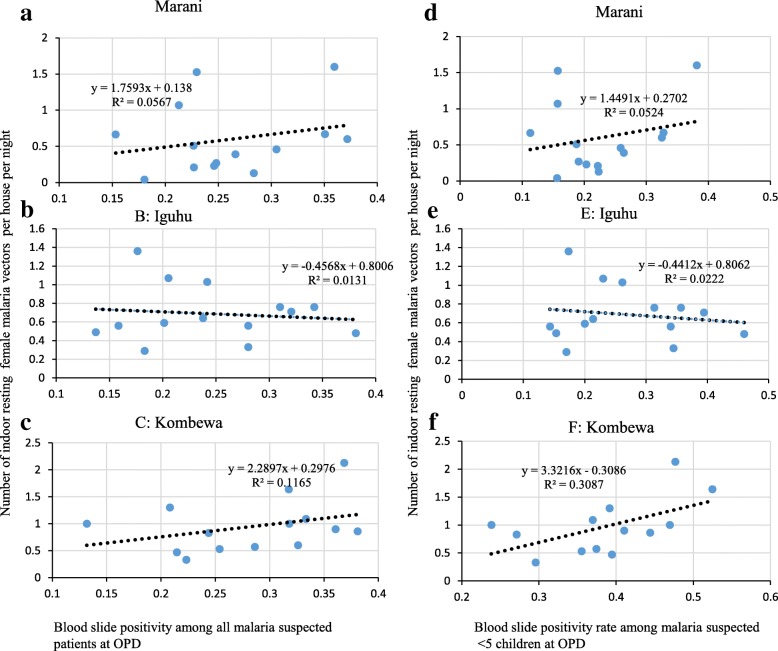
Correlation between monthly abundance of indoor resting female malaria vectors and asymptomatic malaria positivity among school age children in western Kenya from June 2015 to August 2016 (**a**, **b**, **c** show correlation between all age groups OPD malaria positivity and abundance of indoor resting malaria vectors; **d**, **e**, **f** show correlation between OPD malaria positivity among underfive children and abundance of indoor resting malaria vectors

All study sites showed no significant correlation between the abundance of indoor resting vectors (with and without month lag) and OPD malaria positivity (Fig. 4a, b, c, d, e). However, the 1 month lag abundance of vectors was marginally not correlated with OPD positivity among < 5 at Kombewa (rho = 0.51, *p* = 0.08) (Fig. 4f).

### Household survey of anti-malarial drug use

About half of respondents from the high transmission study site (Kombewa), three quarters from the moderate transmission (Iguhu) and as low as 10% from the low transmission setting (Marani) practiced self-malaria treatment (Table 2).

**Table 2:** Household survey on sources of anti-malarial drugs in areas with different infection transmission intensity in western Kenya in June and December 2016

Source of antimalarial drugs	Study sites					
	Marani		Iguhu		Kombewa	
	June 2016	December 2016	June 2016	December 2016	June 2016	December 2016
< 5 age group						
Hospital	99 (91.7%)	43 (93.5%)	93 (88.6%)	18 (66.7%)	76 (91.6%)	34 (44.7%)
Drug shop	09 (8.3%)	03 (6.5%)	12 (11.4%)	09 (32.3%)	7 (8.4%)	42 (55.3%)
5–14 age group						
Hospital	163 (91.5%)	77 (98.7%)	134 (72.8%)	62 (83.8%)	125 (69%)	47 (37.9%)
Drug shop	15 (8.5%)	01 (1.3%)	50 (27.2%)	12 (16.2%)	56 (31%)	77 (60.1%)
≥15 age group						
Hospital	177 (82.7%)	78 (94.0%)	119 (56.4%)	55 (53.9%)	88 (37.2%)	62 (42.5%)
Drug shop	37 (7.3%)	05 (6.0%)	92 (43.6)	47 (46.1%)	148 (62.8%)	84 (57.5%)
Total	500	207	500	203	500	346
Hospital	439 (87.8%)	198 (95.7%)	346 (69.2%)	135 (66.5%)	289 (57.8%)	143 (41.3%)
Drug shop	61 (12.2%)	09 (4.3%)	154 (30.8%)	68 (33.3%)	211 (42.2%)	203 (58.7%)
Proportion of malaria self-treatment	8.25%		32.05%		50.45%	

### Accuracy of blood slide examination

A selected health facility located in the moderate malaria transmission setting revealed an agreement of 88% (175/199) of all examined blood slides between the hospital results and those by two microscopy technicians at the Research Centre (kappa = 0.703; *p* = 0.000). Therefore, a substantial agreement between the hospital blood slide examination results and that at the research Centre observed.

## Discussion

Accuracy of passively collected malaria data in health facilities as a true reflection of infection transmission dynamics in the surrounding communities may be affected by a number of factors including diagnostic and reporting accuracy as well as health seeking behaviour [2, 21–23]. This study aimed at determining the trustworthiness of passive surveillance of malaria in health facilities as a proxy to infection transmission of the catchment community. This study therefore analysed OPD malaria positivity rates and the asymptomatic infection positivity among school children residing in the hospital catchment area.

Approximately one out of four malaria suspected clients examined by blood slide at OPD in three hospitals in western Kenya were malaria parasite positive. Nevertheless, about half of the school children [5–14] examined at the OPD in the low and high infection transmission settings were blood slide positive. This particular age group consistently presented with the highest positivity rate as compared to other age groups and the highest blood slide positivity was in September 2015 at Marani Hospital where two-third among those (5–14 years) suspected of having malaria were blood slide positive. A study in Manicaland province in Zimbabwe reported a higher (48%) positivity rate among all outpatient suspected cases in 2014 [24], this area experienced malaria resurgence. In the Lake endemic setting of Kenya in 2016, malaria accounted for more than 35% OPD of all OPD cases of which also higher than that observed in this study [5]. The study presented existence of higher clinical malaria vulnerability among the 5–14 children in all study areas and among under-fives in high transmission settings. This is due to the fact that under-five children got low sporozoite challenge due to high utilization of bed nets along with their mothers and therefore remain unchallenged throughout their childhood [2]. However, low utilization of LLINs in context of changing infection transmission dynamics this group may be seriously affected because of their weak diseases specific sub-immunity especially in the epidemic prone areas [2, 25–27]. The recent Kenya malaria indicators survey also revealed highest asymptomatic infection susceptibility in this particular age group and therefore proved that the 5–14 age group may reliably be used to monitor transmission dynamics [2]. The highest registered OPD positivity at Marani in September 2015 was preceded by an increased indoor resting vectors as well as highest prevalence of asymptomatic infection among same age group. The observed high blood slide positivity rate could be explained by existence of an outbreak during the study period [6].

The asymptomatic infection among the school children varied from very low at Marani (epidemic prone) to highest at Kombewa (hyperendemic area). Kombewa registered highest prevalence of asymptomatic infection (55%) in June 2015 and lowest in May 2016 after bed nets mass distribution in December 2015. Elsewhere, increasing malaria susceptibility among the school children has been also reported [25, 27, 28].

This study found a significant correlation between asymptomatic malaria positivity among the children and OPD positivity of all age groups at the low transmission setting. Whereas at the moderate transmission study site, the correlation was only observed with the all age groups OPD positivity and < 5 children but not with ≥15 years age group. The correlation between school children asymptomatic malaria positivity and OPD positivity rates for all age groups at the low transmission setting occurred due to fact that clinical malaria susceptibility was higher for all age groups in low transmission settings. Therefore, likelihood of seeking for medical attention is higher as compared to the high transmission settings [26]. Indeed, utilization of health facilities for malaria treatment was higher among all age groups in the low transmission. The absence of correlation with the ≥15 years in moderate transmission settings can also be explained by their low utilization of health facilities. The absence correlation between the asymptomatic infection positivity among school children and the clinical positivity in high transmission settings may be explained also by low utilization of health facilities when seeking for medical attention. This could be due to their low susceptibility to severe form of malaria as compared to their counterparts in highland low transmission settings [2].

In Uganda, a non-linear relationship was observed between clinical malaria attack rate and community asymptomatic malaria positivity in a defined population [29]. In Lake Victoria basin of Tanzania (holoendemic), the OPD positivity was found to be correlated with the asymptomatic malaria positivity among pregnant women and < 5 children attending reproductive and child health clinics [15]. Therefore, absence of the expected correlation between infection dynamics in school age and OPD positivity in high transmission setting could be indeed due to low utilization of health facilities. Moreover, the Kenya malaria indicators survey (2015) also found better health care seeking among febrile children in epidemic prone areas than the Lake endemic area [2]. So poor utilization of health facilities could be a possible reason for this observation. Furthermore, in the moderate transmission study areas where children over also practice self-malaria treatment, the positivity among 5–14 years and the ≥5 years at the OPD showed no correlation with asymptomatic infection positivity among school children. Lack of correlation among these age groups at this site was also due to poor health seeking behaviour. Also there was lower clinical disease susceptibility among the over fives as they were exposed to a significant sporozoite challenge when transmission intensity was still high few years back [4, 6, 7].

Immunoepidemiology of malaria indicated that, population in high transmission setting carry high burden of asymptomatic malaria and only a proportion of the population develops clinical disease, mainly children under-five because of their high exposure to the parasites compared to population in epidemic prone areas [2, 16]. In this areas, majority of the population remains at high risk of ending up with clinical disease and are likely to adhere to treatment and attending health facilities. Also, the older members of the community in epidemic prone areas understand the impact of malaria epidemics, thus, in case they have a suspected malaria cases in the house, are likely to run to health facilities [2]. Also, the impact of public health education on malaria offered during the past malaria epidemics could have an influence the health seeking behaviour.

The population of indoor resting malaria vector collected by PSC was not correlated with the OPD malaria blood slide positivity. This could be due to the reported increasing outdoor resting behaviour, outdoor biting behaviour and changing host preference of previously known anthropophilic vectors [30–34]. Studies from western Kenya shows that more than 90% of indoor host seeking vectors escape through windows and heaves [30]. Moreover presence of resistant knockdown insecticide resistance gene determines on whether the vectors exhibit exophilly or endophilly [35]. The sampled indoor resting vector collected by PSC may therefore not show a relationship with infection transmission rate but may still be used to show trends of vector abundance [7].

This study however had some limitation including lack of a defined catchment area of the selected hospitals. Possibly some patients came from different areas with different infection transmission intensity and thus might have affected the outcome of interest especially in relatively less populated areas. The correction of variables could have been affected by accuracy of microscopic examinations. However, the accuracy of malaria diagnosis has greatly improved over the recent years compared to the past [22]. This has been attributed by continuing on job training and the general malaria diagnostic capacity building [19].

## Conclusion

The hospital based malaria surveillance in low transmission settings strongly correlated with the asymptomatic infection transmission dynamics among school children. Only the ≤5 children OPD positivity correlated significantly with the asymptomatic infection among school age living in mesoendemic study area. No correlation observed between hospital OPD malaria positivity and asymptomatic malaria positivity at the endemic high transmission study site. The use of hospital malaria data in endemic setting as a reflection of the surrounding catchment area may be unreliable despite provision of free anti-malarial drugs at health facilities. Passive and active surveillance of clinical and asymptomatic malaria should be emphasized in endemic settings of Kenya especially in these moments when there are reports of changing infection transmission risks.

## Additional files

Additional file 1: Monthly malaria outpatient positivity rates of the three study sites that are located in areas with different malaria infection transmission intensity in western Kenya from June 2015 to August 2018 (PPTX 306 kb).

Additional file 2: Dynamics of asymptomatic malaria positivity and abundance of indoor resting malaria vectors from three study sites that are located in areas with different malaria infection transmission intensity in western Kenya from June 2015 to August 2016 (PPTX 243 kb).

## References

[1] WHO. World malaria report 2015. Available: http://www.whoint/malaria/publications/world_malaria_report_2015/ Accessed 20 Feb 2018.

[2] National Malaria Control Programme (NMCP), Kenya National Beural of Statistics (KNBS), ICF International (2016). Kenya Malaria indicator Survey 2015.

[3] Mutuku FM, King CH, Mungai P, Mbogo C, Mwangangi J, Muchiri EM (2011). Impact of insecticide-treated bed nets on malaria transmission indices on the south coast of Kenya. Malar J.

[4] Ototo EN, Zhou G, Kamau L, Mbugi JP, Wanjala CL, Machani M (2017). Age-specific Plasmodium parasite profile in pre and post ITN intervention period at a highland site in western Kenya. Malar J.

[5] Machini B, Waqo E, Kizito W, Edwards J, Owiti P, Takarinda K (2016). Trends in outpatient malaria cases, following mass long lasting insecticidal nets (LLIN) distribution in epidemic prone and endemic areas of Kenya. East Afr Med J.

[6] Kapesa A, Kweka JE, Atieli HE, Kamugisha E, Zhou G, Githeko AK, Yan G (2017). Why some sites are responding better to antimalarial interventions? A case study from western Kenya. Malar J.

[7] Zhou G, Lee M-C, Githeko AK, Atieli HE, Yan G (2016). Insecticide-treated net campaign and malaria transmission in western Kenya: 2003–2015. Front Public Health.

[8] Mafigiri R, Matovu JK, Makumbi FE, Ndyanabo A, Nabukalu D, Sakor M (2017). HIV prevalence and uptake of HIV/AIDS services among youths (15–24 years) in fishing and neighboring communities of Kasensero, Rakai District, south western Uganda. BMC Public Health.

[9] Jagannathan P, Muhindo MK, Kakuru A, Arinaitwe E, Greenhouse B, Tappero J (2012). Increasing incidence of malaria in children despite insecticide-treated bed nets and prompt anti-malarial therapy in Tororo, Uganda. Malar J.

[10] Wotodjo AN, Doucoure S, Gaudart J, Diagne N, Sarr FD, Faye N (2017). Malaria in Dielmo, a Senegal village: is its elimination possible after seven years of implementation of long-lasting insecticide-treated nets?. PLoS One.

[11] Tanzania Bureau of Statistics. AIDS and malaria indicator survey 2011–12. Dar es salaam: Tanzania commission for. AIDS. 2013; Available at https://dhsprogram.com/pubs/pdf/AIS11.pd. Accessed 19 Jan 2018.

[12] Kenya Ministry of Health (2014). Kenya Malaria strategy 2009–2018: National Malaria Control Progarm.

[13] Brooker S, Kolaczinski JH, Gitonga CW, Noor AM, Snow RW (2009). The use of schools for malaria surveillance and programme evaluation in Africa. Malar J.

[14] Stevenson JC, Stresman GH, Gitonga CW, Gillig J, Owaga C, Marube E (2013). Reliability of school surveys in estimating geographic variation in malaria transmission in the western Kenyan highlands. PLoS One.

[15] Willilo RA, Molteni F, Mandike R, Mugalura FE, Mutafungwa A, Thadeo A (2016). Pregnant women and infants as sentinel populations to monitor prevalence of malaria: results of pilot study in Lake zone of Tanzania. Malar J.

[16] Zhou G, Afrane YA, Vardo-Zalik AM, Atieli H, Zhong D, Wamae P (2011). Changing patterns of malaria epidemiology between 2002 and 2010 in western Kenya: the fall and rise of malaria. PLoS One.

[17] Ministry of Health (2011). 2010 Malaria indicator survey.

[18] WHO (1975). Manual on practical entomology in malaria. Part II. Methods and techniques.

[19] Odhiambo F, Buff AM, Moranga C, Moseti CM, Wesongah JO, Lowther SA (2017). Factors associated with malaria microscopy diagnostic performance following a pilot quality-assurance programme in health facilities in malaria low-transmission areas of Kenya. 2014 Malaria J.

[20] Gillies M, Coetzee M. A supplement to the Anophelinae of Africa south of the Sahara (Afrotropical region). 1987.

[21] Metta E, Haisma H, Kessy F, Hutter I, Bailey A (2014). “We have become doctors for ourselves”: motives for malaria self-care among adults in southeastern Tanzania. Malar J.

[22] Afrane YA, Zhou G, Githeko AK, Yan G (2013). Utility of health facility-based malaria data for malaria surveillance. PLoS One.

[23] Kunimitsu A (2009). The accuracy of clinical malaria case reporting at primary health care facilities in Honiara, Solomon Islands. Malar J.

[24] Mutsigiri F, Mafaune PT, Mungati M, Shambira G, Bangure D, Juru T (2017). Malaria morbidity and mortality trends in Manicaland province, Zimbabwe, 2005-2014. Pan African Med J.

[25] Mathanga DP, Halliday KE, Jawati M, Verney A, Bauleni A, Sande J (2015). The high burden of malaria in primary school children in southern Malawi. Am. J. Trop. Med. Hyg..

[26] Wanjala CL, Waitumbi J, Zhou G, Githeko AK (2011). Identification of malaria transmission and epidemic hotspots in the western Kenya highlands: its application to malaria epidemic prediction. Parasit Vectors.

[27] Kepha S, Nikolay B, Nuwaha F, Mwandawiro CS, Nankabirwa J, Ndibazza J (2016). Plasmodium falciparum parasitaemia and clinical malaria among school children living in a high transmission setting in western Kenya. Malar J.

[28] O'Meara WP, Bejon P, Mwangi TW, Okiro EA, Peshu N, Snow RW (2008). Effect of a fall in malaria transmission on morbidity and mortality in Kilifi, Kenya. Lancet.

[29] Boyce RM, Reyes R, Matte M, Ntaro M, Mulogo E, Lin F-C (2016). Practical implications of the non-linear relationship between the test positivity rate and malaria incidence. PLoS One.

[30] Ndenga BA, Mulaya NL, Musaki SK, Shiroko JN, Dongus S, Fillinger U (2016). Malaria vectors and their blood-meal sources in an area of high bed net ownership in the western Kenya highlands. Malar J.

[31] Degefa T, Yewhalaw D, Zhou G, Lee M-C, Atieli H, Githeko AK (2017). Indoor and outdoor malaria vector surveillance in western Kenya: implications for better understanding of residual transmission. Malar J.

[32] Ototo EN, Mbugi JP, Wanjala CL, Zhou G, Githeko AK, Yan G (2015). Surveillance of malaria vector population density and biting behaviour in western Kenya. Malar J.

[33] Wamae P, Githeko A, Otieno G, Kabiru E, Duombia S (2015). Early biting of the Anopheles gambiae ss and its challenges to vector control using insecticide treated nets in western Kenya highlands. Acta Trop.

[34] Cooke MK, Kahindi SC, Oriango RM, Owaga C, Ayoma E, Mabuka D (2015). A bite before bed’: exposure to malaria vectors outside the times of net use in the highlands of western Kenya. Malar J.

[35] Githinji E, Irungu L, Ndegwa P, Atieli F, Machani M (2017). Effects of kdr gene frequencies on major malaria vectors’ resting behaviour in Teso sub-counties, western Kenya. The Kash 7 abstract submission.

