# Supplementary material for: Utility of passive malaria surveillance in hospitals as a surrogate to community infection transmission dynamics in western Kenya
Source: Arch Public Health. 2018 Jul 26;76:39. doi: 10.1186/s13690-018-0288-y (PMC6060476; doi:10.1186/s13690-018-0288-y)
Supplement: Supplementary file 1 — Monthly malaria outpatient positivity rates of the three study sites that are located in areas with different malaria infection transmission intensity in western Kenya from June 2015 to August 2018 (PPTX 306 kb). [file 13690_2018_288_MOESM1_ESM.pptx]

## Slide 1
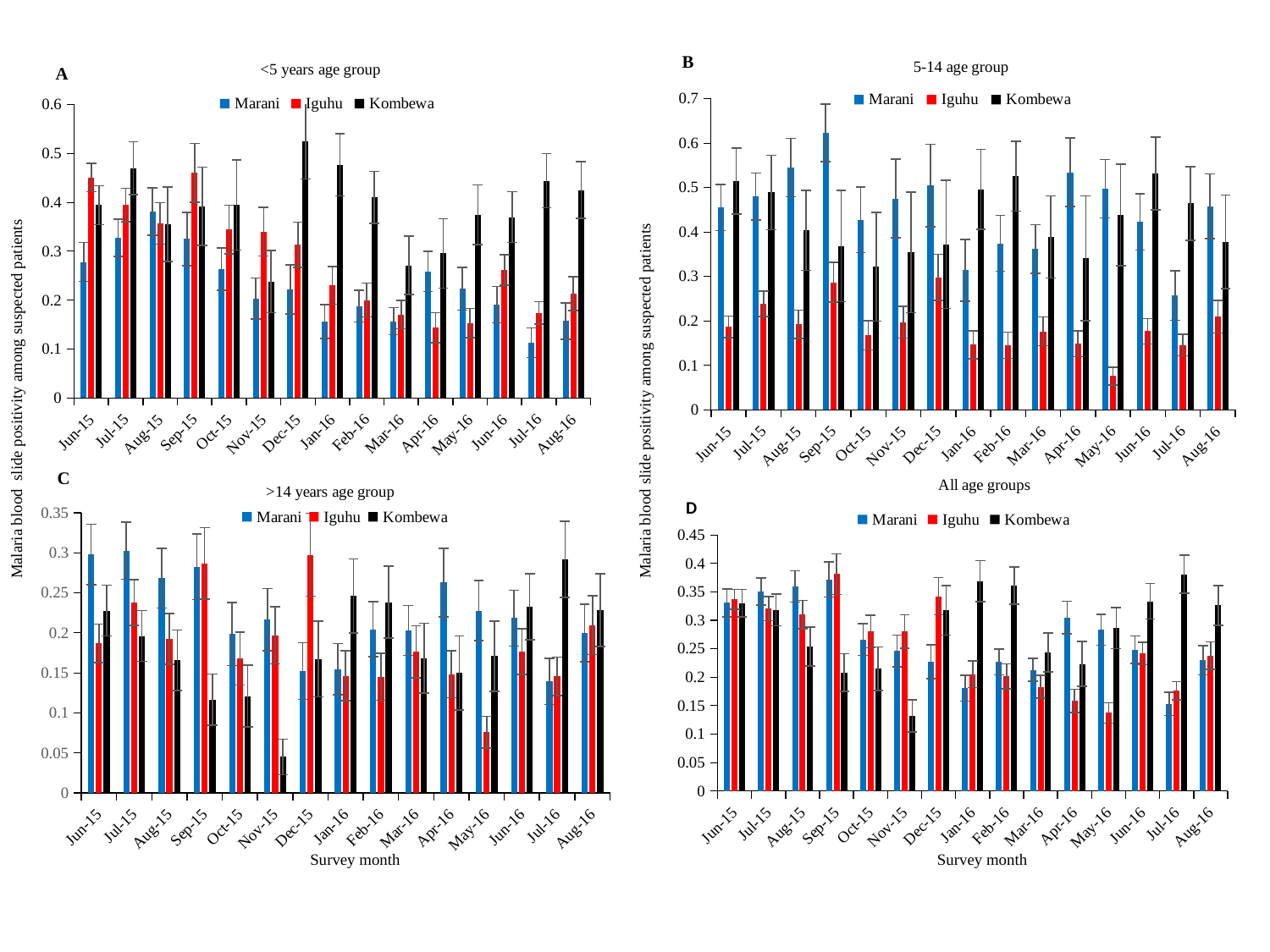

### Chart: 5-14 age group
| Category | Marani | Iguhu | Kombewa |
|---|---|---|---|
| 42156 | 0.4548022598870056 | 0.18693467336683417 | 0.5144508670520231 |
| 42186 | 0.4796511627906977 | 0.23798358733880423 | 0.48905109489051096 |
| 42217 | 0.545045045045045 | 0.19230769230769232 | 0.40350877192982454 |
| 42248 | 0.6232558139534884 | 0.2868020304568528 | 0.3684210526315789 |
| 42278 | 0.4277456647398844 | 0.16768916155419222 | 0.32142857142857145 |
| 42309 | 0.47540983606557374 | 0.1970649895178197 | 0.3541666666666667 |
| 42339 | 0.5045045045045045 | 0.2976588628762542 | 0.37209302325581395 |
| 42370 | 0.313953488372093 | 0.14634146341463414 | 0.4957983193277311 |
| 42401 | 0.3744493392070485 | 0.14492753623188406 | 0.5256410256410257 |
| 42430 | 0.36177474402730375 | 0.17636022514071295 | 0.3888888888888889 |
| 42461 | 0.5341614906832298 | 0.14840989399293286 | 0.3409090909090909 |
| 42491 | 0.4977578475336323 | 0.07591240875912408 | 0.4383561643835616 |
| 42522 | 0.4230769230769231 | 0.17673048600883653 | 0.5314685314685315 |
| 42552 | 0.25738396624472576 | 0.14580801944106925 | 0.4642857142857143 |
| 42583 | 0.4581005586592179 | 0.2097457627118644 | 0.3780487804878049 |
### Chart: <5 years age group
| Category | Marani | Iguhu | Kombewa |
|---|---|---|---|
| 42156 | 0.27800829875518673 | 0.45065502183406114 | 0.39414802065404475 |
| 42186 | 0.32764505119453924 | 0.3944374209860936 | 0.4696969696969697 |
| 42217 | 0.38144329896907214 | 0.35699797160243407 | 0.35526315789473684 |
| 42248 | 0.325 | 0.4600760456273764 | 0.3916083916083916 |
| 42278 | 0.263681592039801 | 0.3446327683615819 | 0.3944954128440367 |
| 42309 | 0.2033898305084746 | 0.3400576368876081 | 0.23837209302325582 |
| 42339 | 0.22180451127819548 | 0.3134715025906736 | 0.5246913580246914 |
| 42370 | 0.15632183908045977 | 0.2299349240780911 | 0.4767932489451477 |
| 42401 | 0.1875 | 0.2 | 0.41033434650455924 |
| 42430 | 0.15695067264573992 | 0.17024539877300612 | 0.27102803738317754 |
| 42461 | 0.25874125874125875 | 0.14342629482071714 | 0.29559748427672955 |
| 42491 | 0.22318840579710145 | 0.15342960288808663 | 0.37448559670781895 |
| 42522 | 0.19080459770114944 | 0.2614980289093298 | 0.3696969696969697 |
| 42552 | 0.11294117647058824 | 0.1737891737891738 | 0.4440894568690096 |
| 42583 | 0.15718157181571815 | 0.21348314606741572 | 0.425 |Malaria blood slide positivity among suspected patients
Malaria blood slide positivity among suspected patients
### Chart: >14 years age group
| Category | Marani | Iguhu | Kombewa |
|---|---|---|---|
| 42156 | 0.2976827094474153 | 0.18693467336683417 | 0.22781065088757396 |
| 42186 | 0.30265210608424337 | 0.23798358733880423 | 0.19601328903654486 |
| 42217 | 0.26838235294117646 | 0.19230769230769232 | 0.16576086956521738 |
| 42248 | 0.2826086956521739 | 0.2868020304568528 | 0.11658031088082901 |
| 42278 | 0.1984924623115578 | 0.16768916155419222 | 0.12087912087912088 |
| 42309 | 0.21627906976744185 | 0.1970649895178197 | 0.04491017964071856 |
| 42339 | 0.15228426395939088 | 0.2976588628762542 | 0.16736401673640167 |
| 42370 | 0.15478615071283094 | 0.14634146341463414 | 0.24624624624624625 |
| 42401 | 0.2045889101338432 | 0.14492753623188406 | 0.23837209302325582 |
| 42430 | 0.203125 | 0.17636022514071295 | 0.16842105263157894 |
| 42461 | 0.2628992628992629 | 0.14840989399293286 | 0.14977973568281938 |
| 42491 | 0.2277432712215321 | 0.07591240875912408 | 0.1708185053380783 |
| 42522 | 0.21851851851851853 | 0.17673048600883653 | 0.2325 |
| 42552 | 0.13949275362318841 | 0.14580801944106925 | 0.29178470254957506 |
| 42583 | 0.2 | 0.2097457627118644 | 0.22865853658536586 |
### Chart: All age groups
| Category | Marani | Iguhu | Kombewa |
|---|---|---|---|
| 42156 | 0.33070866141732286 | 0.33671252715423605 | 0.3300699300699301 |
| 42186 | 0.35073201782304264 | 0.3206314643440392 | 0.31805425631431244 |
| 42217 | 0.3596187175043328 | 0.3101361573373676 | 0.2539432176656151 |
| 42248 | 0.3717277486910995 | 0.3812233285917496 | 0.20819112627986347 |
| 42278 | 0.26618705035971224 | 0.28024819027921405 | 0.2146118721461187 |
| 42309 | 0.24613686534216336 | 0.2803532008830022 | 0.13176895306859207 |
| 42339 | 0.22697795071335927 | 0.3423312883435583 | 0.31756756756756754 |
| 42370 | 0.18032786885245902 | 0.20535714285714285 | 0.3686502177068215 |
| 42401 | 0.2267175572519084 | 0.2012728719172633 | 0.3606755126658625 |
| 42430 | 0.2128589263420724 | 0.1829608938547486 | 0.24382207578253706 |
| 42461 | 0.3049147442326981 | 0.15840779853777417 | 0.22325581395348837 |
| 42491 | 0.28353948620361563 | 0.13714679531357685 | 0.2864321608040201 |
| 42522 | 0.24813895781637718 | 0.24178272980501392 | 0.3333333333333333 |
| 42552 | 0.15321252059308071 | 0.17633726201269265 | 0.38089330024813894 |
| 42583 | 0.22971652003910067 | 0.23793395427603725 | 0.32608695652173914 |D
Survey month
Survey month
