# Supplementary material for: Utility of passive malaria surveillance in hospitals as a surrogate to community infection transmission dynamics in western Kenya
Source: Arch Public Health. 2018 Jul 26;76:39. doi: 10.1186/s13690-018-0288-y (PMC6060476; doi:10.1186/s13690-018-0288-y)
Supplement: Supplementary file 2 — Dynamics of asymptomatic malaria positivity and abundance of indoor resting malaria vectors from three study sites that are located in areas with different malaria infection transmission intensity in western Kenya from June 2015 to August 2016 (PPTX 243 kb). [file 13690_2018_288_MOESM2_ESM.pptx]

## Slide 1
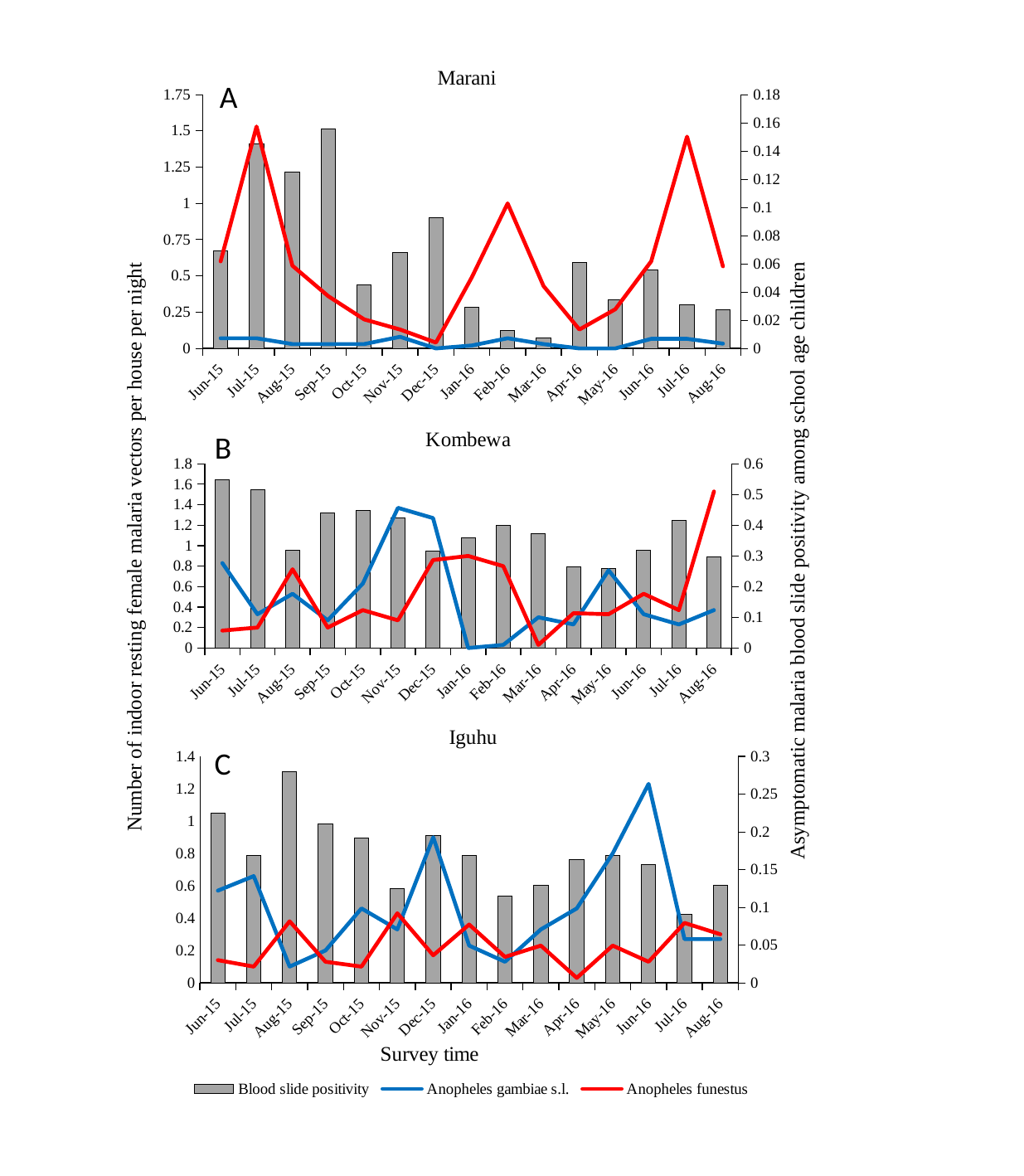

### Chart: Marani
| Category | Blood slide positivity | Female Anopheles gambiae | Female Anopheles funestus |
|---|---|---|---|
| 42156 | 0.06930693069306931 | 0.07 | 0.6 |
| 42186 | 0.1450381679389313 | 0.07 | 1.53 |
| 42217 | 0.125 | 0.03 | 0.57 |
| 42248 | 0.15584415584415584 | 0.03 | 0.36 |
| 42278 | 0.045112781954887216 | 0.03 | 0.2 |
| 42309 | 0.06779661016949153 | 0.08 | 0.13 |
| 42339 | 0.09259259259259259 | 0.0 | 0.04 |
| 42370 | 0.02912621359223301 | 0.02 | 0.49 |
| 42401 | 0.012987012987012988 | 0.07 | 1.0 |
| 42430 | 0.007633587786259542 | 0.03 | 0.43 |
| 42461 | 0.061068702290076333 | 0.0 | 0.13 |
| 42491 | 0.034722 | 0.0 | 0.27 |
| 42522 | 0.05555 | 0.066 | 0.6 |
| 42552 | 0.031 | 0.066 | 1.46 |
| 42583 | 0.02721 | 0.033 | 0.566 |A
Number of indoor resting female malaria vectors per house per night
Asymptomatic malaria blood slide positivity among school age children
### Chart: Kombewa
| Category | Blood slide positivity | Female Anopheles gambiae | Female Anopheles Funestus |
|---|---|---|---|
| 42156 | 0.5492957746478874 | 0.83 | 0.17 |
| 42186 | 0.5149700598802395 | 0.33 | 0.2 |
| 42217 | 0.3181818181818182 | 0.53 | 0.77 |
| 42248 | 0.44036697247706424 | 0.27 | 0.2 |
| 42278 | 0.4476744186046512 | 0.63 | 0.37 |
| 42309 | 0.4247787610619469 | 1.37 | 0.27 |
| 42339 | 0.31496062992125984 | 1.27 | 0.86 |
| 42370 | 0.36 | 0.0 | 0.9 |
| 42401 | 0.3984375 | 0.03 | 0.8 |
| 42430 | 0.3728813559322034 | 0.3 | 0.03 |
| 42461 | 0.263157894736842 | 0.23 | 0.34 |
| 42491 | 0.2583 | 0.76 | 0.33 |
| 42522 | 0.3185 | 0.33 | 0.53 |
| 42552 | 0.41509 | 0.23 | 0.37 |
| 42583 | 0.298 | 0.37 | 1.53 |B
### Chart: Iguhu
| Category | Blood slide positivity | Anopheles gambiae s.l. | Anopheles funestus |
|---|---|---|---|
| 42156 | 0.225 | 0.57 | 0.14 |
| 42186 | 0.16826923076923078 | 0.66 | 0.1 |
| 42217 | 0.28 | 0.1 | 0.38 |
| 42248 | 0.21052631578947367 | 0.2 | 0.13 |
| 42278 | 0.19170984455958548 | 0.46 | 0.1 |
| 42309 | 0.125 | 0.33 | 0.43 |
| 42339 | 0.1949685534591195 | 0.9 | 0.17 |
| 42370 | 0.16875 | 0.23 | 0.36 |
| 42401 | 0.1144578313253012 | 0.13 | 0.16 |
| 42430 | 0.12921348314606743 | 0.33 | 0.23 |
| 42461 | 0.16363636363636364 | 0.46 | 0.03 |
| 42491 | 0.1687 | 0.8 | 0.23 |
| 42522 | 0.156626 | 1.23 | 0.13 |
| 42552 | 0.09036 | 0.27 | 0.37 |
| 42583 | 0.12941 | 0.27 | 0.3 |C
